# NOS1 inhibits the interferon response of cancer cells by S-nitrosylation of HDAC2

**DOI:** 10.1186/s13046-019-1448-9

**Published:** 2019-12-05

**Authors:** Pengfei Xu, Shuangyan Ye, Keyi Li, Mengqiu Huang, Qianli Wang, Sisi Zeng, Xi Chen, Wenwen Gao, Jianping Chen, Qianbing Zhang, Zhuo Zhong, Ying Lin, Zhili Rong, Yang Xu, Bingtao Hao, Anghui Peng, Manzhao Ouyang, Qiuzhen Liu

**Affiliations:** 1 Cancer Research Institute, Guangdong Provincial Key Laboratory of Cancer Immunotherapy, Guangzhou key laboratory of tumor immunology research, School of Basic Medical Sciences, Southern Medical University, Guangzhou, 510515 China; 2 Department of Oncology, Guangzhou Hospital of Integrated Traditional and Western Medicine, Guangzhou, 510800 China; 3 Center for medical transformation, Shunde Hospital, Southern Medical University, Foshan, 528308 China

**Keywords:** NOS1, S-nitrosylation, Melanoma, HDAC2, IFNα, H4K16ac, Metastasis

## Abstract

**Background:**

The dysfunction of type I interferon (IFN) signaling is an important mechanism of immune escape and metastasis in tumors. Increased NOS1 expression has been detected in melanoma, which correlated with dysfunctional IFN signaling and poor response to immunotherapy, but the specific mechanism has not been determined. In this study, we investigated the regulation of NOS1 on the interferon response and clarified the relevant molecular mechanisms.

**Methods:**

After stable transfection of A375 cells with NOS1 expression plasmids, the transcription and expression of IFNα-stimulated genes (ISGs) were assessed using pISRE luciferase reporter gene analysis, RT-PCR, and western blotting, respectively. The effect of NOS1 on lung metastasis was assessed in melanoma mouse models. A biotin-switch assay was performed to detect the S-nitrosylation of HDAC2 by NOS1. ChIP-qPCR was conducted to measure the binding of HDAC2, H4K16ac, H4K5ac, H3ac, and RNA polymerase II in the promoters of ISGs after IFNα stimulation. This effect was further evaluated by altering the expression level of HDAC2 or by transfecting the HDAC2-C262A/C274A site mutant plasmids into cells. The coimmunoprecipitation assay was performed to detect the interaction of HDAC2 with STAT1 and STAT2. Loss-of-function and gain-of-function approaches were used to examine the effect of HDAC2-C262A/C274A on lung metastasis. Tumor infiltrating lymphocytes were analyzed by flow cytometry.

**Results:**

HDAC2 is recruited to the promoter of ISGs and deacetylates H4K16 for the optimal expression of ISGs in response to IFNα treatment. Overexpression of NOS1 in melanoma cells decreases IFNα-responsiveness and induces the S-nitrosylation of HDAC2-C262/C274. This modification decreases the binding of HDAC2 with STAT1, thereby reducing the recruitment of HDAC2 to the ISG promoter and the deacetylation of H4K16. Moreover, expression of a mutant form of HDAC2, which cannot be nitrosylated, reverses the inhibition of ISG expression by NOS1 in vitro and decreases NOS1-induced lung metastasis and inhibition of tumor infiltrating lymphocytes in a melanoma mouse model.

**Conclusions:**

This study provides evidence that NOS1 induces dysfunctional IFN signaling to promote lung metastasis in melanoma, highlighting NOS1-induced S-nitrosylation of HDAC2 in the regulation of IFN signaling via histone modification.

## Background

Type I interferon (IFN) plays a pivotal role in suppressing neoplastic growth and shaping tumor immunogenicity. Both IFNs produced by malignant cells and tumor-infiltrating dendritic cells may underlie cancer immunosurveillance [1]. Tumor cells express type I IFN receptors and can produce IFNs, which not only optimally activate the antitumor response in immune cells but also directly induce the expression of tumor antigens and affect tumor cell growth, survival, and sensitivity to some chemical treatments [1, 2]. Dysfunction in IFN signaling is involved in tumorigenesis, tumor progression and cancer immune escape [3, 4]. In addition, the therapeutic effects of chemotherapeutic agents, targeted anticancer agents, are largely dependent on intact type I IFN signaling in cancer cells [5]. The intratumoural expression levels of IFNs or of IFN-stimulated genes (ISGs) correlate with favorable disease outcome [6, 7]. In contrast, the absence of type I IFN signaling leads to rapid tumor growth and shortened survival in animal models [8]. Moreover, evidence has indicated that restoration of IFN signaling in breast cancer cells leads to reduced bone metastasis and prolonged survival time [9]. Recently, two studies have also indicated a key role of the functional IFN pathway in melanoma patients for sensitivity to PD-1 or CTLA-4 blockade immunotherapy [10, 11]. These studies highlight the critical role of IFN signaling in melanoma cell immune surveillance, consistent with the dysregulation of the IFN signaling pathway that promotes melanoma progression. Considering the prevalence of nonresponse of IFNα in melanoma cells and tissues [12, 13], uncovering the mechanism of IFN dysfunction may be helpful for improving the therapeutic effect of the IFNα-based approach and improving the efficacy of chemotherapy and immunotherapy for tumor control in patients.

The biological effects of IFNs are mediated by signaling through IFN receptors and the activation of ISGs that encode effector proteins. IFNα binding to its transmembrane receptor induces the phosphorylation of STAT1 and STAT2, which, together with IRF9, form the transcription factor complex known as IFN-stimulated gene factor 3 (ISGF3). This complex translocates into the nucleus, and binds to the interferon-sensitive response element (ISRE) sequence of the promoter, leading to the expression of ISGs [14]. Histone modifications emerge as critical mechanisms for the regulation of IFNα signaling. In contrast to the common role of histone deacetylases (HDACs) in gene repression, HDAC activity provides a required positive function for the IFNα response. Generally, blocking HDAC activity with inhibitors prevents the induction of ISGs and the innate antiviral response [15]. HDAC activity has been found to be required between ISGF3 promoter occupation and RNA polymerase II (RNA pol II) recruitment [15–17]. These observations suggest that the regulatory effect of HDACs on IFN signaling occurs primarily at the transcriptional level. Additionally, previous studies reported that histone H4 becomes deacetylated in ISG54 promoters in response to IFNα, suggesting that it may be a target for HDACs [16]. However, there is no evidence that HDAC directly promotes ISG expression by deacetylating histone H4. Importantly, the contribution of individual HDACs to this phenomenon remained unclear until recently. In particular, HDAC2, a class I HDAC, has been shown to be required for type I and type II IFN signaling [18]. Inhibition of HDAC2 by small interfering RNA (siRNA) decreases IFNα responsiveness, demonstrating that HDAC2 modulates IFNα-induced transcription [19]. Furthermore, the Sin3A complex that interacts with HDAC2 primarily inhibits transcription but is required for ISG transcriptional elongation [20]. Nevertheless, the transcriptional regulation of ISG is a rather complex and dynamic process, and the mechanisms governing this process have not been thoroughly elucidated to date.

Nitric oxide, a signaling molecule synthesized by three isoforms of NO synthase (NOS1, NOS2 and NOS3), increases in multiple cancers and participates in various cancer processes such as formation, progression and metastasis [21]. Investigations have demonstrated multiple roles for NO in melanoma pathology, and elevated levels of NO prognosticate a poor outcome for melanoma patients [22]. NO/NOSs primarily participate in the regulation of cellular function, and gene transcription through important target molecules of S-nitrosylation modification [23]. Numerous S-nitrosylated proteins have been reported to be involved in various cancer-related events, such as p53, PTEN, Bcl-2, Caspases, and EGFR [24]. Our previous studies demonstrated that NOS1 was highly expressed in melanoma cells and involved in inhibiting the reactivity of PBMCs to IFNα, revealing the critical role of NOS1 in tumor immune escape, but the specific mechanism governing this role has not been determined [25]. NOS1 can selectively induce S-nitrosylation of HDAC2 at specific cysteine residues (Cys-262, Cys-274). S-nitrosylation of HDAC2 does not affect deacetylase activity but inhibits its association with target genes, which leads to chromatin remodeling during neuronal development, thereby promoting dendritic growth and branching via CREB activation [26]. Whether NOS1 participates in the regulation of the IFNα response through S-nitrosylation of HDAC2 in tumor cells has not been determined. To address this question, we investigated the regulation of NOS1 on the interferon response and clarified the relevant molecular mechanisms, which suggested a new means of targeting NOS1 in the treatment of melanoma.

## Materials and methods

### Cell line culture, plasmids, antibodies, and reagents

The human melanoma cell lines A375, human colorectal cancer cell lines SW480, human ovarian cancer cell lines SKOV3 and mouse melanoma cell lines B16F10 were purchased from American Type Cell Collection (ATCC, Manassas, VA, USA). Cells were maintained in DMEM or RPMI 1640 (Gibco, Gaithersburg, MD, USA) supplemented with 10% fetal bovine serum (BI, Salt Lake City, UT, USA) and 1% penicillin/streptomycin solution in a humidified 37 **°**C incubator. Stable NOS1 overexpression and nontargeted control cell lines were generated according to a previously reported method [27]. pcDNA3.1–HDAC2 (WT, C262A/C274A-MUT, Flag-tagged, human) and pLVX-mCherry-C1-HDAC2 (WT, C262A/C274A-MUT, Flag-tagged, mouse) were designed and synthesized from Synbio Technologies (Suzhou, China). The luciferase reporter plasmids pISRE-TA-luciferase and pRL-SV40-Renilla-luciferase were purchased from Beyotime Biotechnology (Shanghai, China). The chemicals GSNO, N-PLA, L-NAME, and 1400 W were obtained from Cayman Chemical (Ann Arbor, MI, USA). Human and mouse IFNα (Sigma-Aldrich, St. Louis, MO, USA) was used to treat the cells for the indicated duration of time at a concentration of 1000 units per ml. In the in vivo experiment, tumor-bearing mice were given an intraperitoneal injection of 30,000 U/day IFNα 3 times before sacrifice. The primary antibodies against HDAC2, NOS1, STAT1, STAT2 and acetyl histone H4K16 were provided by Cell Signaling Technology (CST, Beverly, MA, USA). Antibodies recognizing acetyl histone H4K5 and Rpb1 were purchased from Abcam (Cambridge, MA, USA). The normal rabbit IgG antibody and acetyl histone H3 antibody were obtained from Millipore (Boston, MA, USA). The anti-GAPDH, anti-Flag, anti-H4, and goat anti-rabbit secondary antibodies were purchased from Proteintech (Wuhan, China). DAPI and Alexa Fluor 488-conjugated goat anti-rabbit antibodies were purchased from Invitrogen (Carlsbad, CA, USA).

### RNA extraction and quantitative PCR (qPCR)

Total RNA was extracted from cultured cells or tumor tissue, and cDNA was synthesized using RNAiso Plus reagent (Takara, Shiga, Japan) and PrimeScript RT kit (Takara), respectively. qPCR was performed on a LightCycler 96 System (Roche Life Science) using TB Green Premix Ex Taq II (Takara) and the primer pairs listed in Additional file 1: Table S1. The reactions were performed in 30 s at 95 °C for initial denaturation and in 5 s at 95 °C, 30 s at 55 °C, and 30 s at 72 °C for 45 cycles. All samples were normalized to the endogenous control GAPDH, and relative fold expression levels were calculated using the 2^−ΔΔCt^ method [28]. All experiments were performed independently at least three times, with all samples being analyzed in triplicate.

### Small interfering RNA (siRNA) and plasmid transfection

The siRNA against HDAC2 and scrambled control sequences were synthesized by Synbio Technologies (Suzhou, China), and are listed in Additional file 1: Table S2. Cells were seeded into 6-well plates (5 × 10^5^/well) and cultured without penicillin and streptomycin overnight. The next day, cells were transfected with Opti-MEM medium (Invitrogen, Gibco, China), lipofectamine 3000 (Invitrogen), 100 nM siRNA or 2 μg plasmids according to the manufacturer’s recommendations. Six hours after transfection, the medium was replaced with fresh growth medium. After culturing for 24–72 h, cells were used for further experiments. RT-PCR and immunoblotting were used to verify the transfection efficiency.

### Western blotting

Total protein was extracted by lysing with RIPA buffer containing PMSF (1 mM) and phosphatase inhibitor (1 mM) mixture. Nuclear and cytoplasmic extracts were prepared using a kit (Cat. No. P0028, Beyotime Biotechnology) according to the manufacturer’s protocol. The expression of each protein was analyzed using western blotting according to a previously reported method [29]. Briefly, 30 μg of protein per well was detached by SDS-PAGE. The sample was transferred to PVDF membranes (Millipore). After blocking with 5% BSA for 1–2 h, the membranes were incubated with the diluted appropriate primary (1:1000) and HRP-conjugated IgG secondary (1:10000) antibodies. Signals were visualized using the ECL Western Blot Kit (Millipore).

### Immunofluorescence assay

Immunofluorescence was performed as previously described [29]. A375 cells were seeded on coverslips and treated with GSNO (100 μM) for 30 min. The cells were then fixed and permeabilized with 4% paraformaldehyde and 0.1% Triton X-100, respectively. After blocking with 5% BSA, cells were probed with the primary anti-HDAC2 antibody overnight at 4 °C and the corresponding Alexa Fluor 488 antibody, followed by counterstaining with DAPI solution. Analysis was performed using a florescence microscope (Nikon Eclipse Ti-U, Japan).

### ChIP-qPCR

Chromatin immunoprecipitation (ChIP) assays were performed using the SimpleChIP Plus Sonication Chromatin IP Kit (Cat. No. 56383, CST) by following the manufacturer’s guidelines. Briefly, A375 cells were fixed with formaldehyde to crosslink DNA and protein, and sonicated to yield 150-bp to 900-bp fragments. The protein-DNA complexes were precipitated using the normal rabbit IgG antibody and polyclonal antibodies. For each immunoprecipitation, 10 μg of antibody was added to the lysate and incubated overnight at 4 °C with rotation. Then, 30 μl of protein G magnetic beads were added and incubated at 4 °C for 2 h with rotation. Precipitin G beads were precipitated and washed sequentially with low-salt and high-salt wash buffer. The protein-DNA complex was reversed at 65 **°**C overnight followed by DNA purification. Enrichment of the DNA sequences was detected using qPCR as described above with the primers listed in Additional file 1: Table S3. The data were normalized and analyzed using the percent input method as follows:

$$ \mathrm{Percent}\ \mathrm{Input}=2\%\times \kern0.37em {2}^{\left(\mathrm{C}\ \left[\mathrm{T}\right]\ 2\%\mathrm{Input}\ \mathrm{Sample}-\mathrm{C}\ \left[\mathrm{T}\right]\ \mathrm{IP}\ \mathrm{Sample}\right)}. $$

$$ \mathrm{C}\ \left[\mathrm{T}\right]=\mathrm{Threshold}\ \mathrm{cycle}\ \mathrm{of}\ \mathrm{PCR}. $$

### Dual-luciferase reporter gene assay

A375 cells were cotransfected with 1 μg of pISRE-TA-luciferase and 0.01 μg of pRL-SV40-Renilla-luciferase plasmids for 24 h using lipofectamine 3000 reagent (Invitrogen), and then treated with or without IFNα for 6 h. A Dual-Luciferase Reporter Gene Assay Kit (Beyotime) was used to measure the luciferase activities. To determine the effect of HDAC2 on IFNα-induced transcriptional activity, reporter genes were cotransfected with plasmids expressing HDAC2 (1 μg) or siRNAs (100 nM) specific for HDAC2. The plasmids and siRNAs were transfected into A375 cells using the method described above. Data were normalized for transfection efficiency by comparing the firefly luciferase (LUC) activity with that of Renilla luciferase (REN).

### Coimmunoprecipitation (co-IP)

Immunoprecipitates were obtained using the Co-Immunoprecipitation Kit (Cat. No.26149, Thermo Fisher), as we described previously [27]. The assay was performed according to standard procedures.

### S-nitrosylation detection assay

S-nitrosylated protein detection assays were performed as described previously [29]. Briefly, 100–250 μg protein lysates were extracted from A375 and SKOV3 cells treated with IFNα (1000 U/ml), GSNO (100 μM), L-NAME (1 mM), N-PLA (100 μM), or 1400 W(100 μM). Biotinylated proteins can be easily detected by biotin western blot or streptavidin precipitation followed by western blotting.

### Histone deacetylase activity assay

For the HDAC2 activity assay, immunoprecipitated proteins were obtained using an IP kit (Cat. No.26149, Thermo Fisher), and then assessed using a fluorogenic HDAC activity assay kit (Cat. No. 13601, AAT Bioquest), as described previously [26]. Briefly, the extracts were transferred to a black 96-well plate and the fluorescence intensity of Ex/Em = 490/525 was monitored using a multifunction microplate reader. All experiments were repeated three times.

### CRISPR-Cas9-mediated genome editing, Lentivirus production and cell line selection

HDAC2-KO cells were obtained using the CRISPR-Cas9 system as described previously [30]. The guide RNA (target sequences: TGAGTCATCCGGATTCTATGAGG) was cloned into the Cas9 vector (NEWMOL, Synbio Technologies). Guide RNA-encoding plasmids were transfected into B16F10 cells for 48 h as described above. Transfected cells were selected with G418 (300 ng/ml) to generate stable clonal lines from single cells, and individual clones were picked and cultured. Gene defects were identified by RT-PCR and immunoblotting. Lentiviral production was performed based on a previously described protocol [31]. Stable HDAC2-WT/MUT expression cell lines were generated by HDAC2-KO cells infected with lentivirus vector encoding HDAC2-WT/MUT. Stable clones were selected with puromycin (1.5 μg/ml).

### Tumor models

All animal experiments in this study were approved by the Medical Ethics Committee of Southern Medical University and conducted in strict accordance with the guidelines from the Ministry of Science and Technology of China. C57BL/6 mice and BALB/c-nu mice (Female, 6–8 weeks old) were all purchased from Guangdong Medical Laboratory Animal Center. To construct a lung metastasis model of melanoma, 1–3 × 10^6^ B16F10 cells were intravenously injected into mice. After cell injection, the mice were randomly assigned to the experimental and control groups (5–14 mice per group), and they were then housed in SPF facilities on a 12-h light/dark cycle until the end of the experiment. Mice were euthanized during days 11–17 postinjection, and lung tissue was isolated, photographed and then fixed with 4% formaldehyde for histological and morphometric measurements. In some cases, mice were sacrificed individually upon signs of metastatic distress and lung metastasis confirmed via histology and lung weight. The number of visible tumors in the lungs was counted separately, and fixed murine lungs were routinely processed and embedded in paraffin. Paraffin sections (5 μm) were stained with H&E according to standard protocols, examined by microscopy and photographed.

### Flow cytometry

For analysis of tumor infiltrating lymphocytes, resected tumor tissues were cut into small pieces and then digested in collagenase I (1 mg/ml) and 13.3 μl DNase I (50 U/ml) at 37 °C for 30 min. The mixture was filtered through a 70-μm strainer to prepare a single cell suspension. Cells were then washed twice with PBS and re-suspended in PBS, and 1 × 10^6^ cells were incubated with 3 μl antibody for 30 min at 4 °C in darkness. Wash the cells twice and perform the analysis on the FACSCalibur (BD Biosciences, USA). The anti-mouse CD3-PE-Cy7, CD8-FITC, CD45-APC-Cy7, F4/80-PE, CD25- PerCP-Cy5.5 and CD11b-BV650 antibodies were all purchased from BD Biosciences. Data are represented as the percentage of lymphocytes as indicated.

### Statistical analysis

Generation of all graphs and statistical analyses was performed with GraphPad Prism 7.0 software (San Diego, California, USA). Each experiment was repeated at least three times independently. The results are expressed as the mean values ± SD; the comparisons between groups were analyzed using Student’s t-test. Kaplan–Meier survival plots were compared using a log-rank test. A *P* value < 0.05 was considered to be statistically significant.

## Results

### NOS1 blocks IFNα-stimulated gene induction and promotes lung metastasis of melanoma

**Fig. 1:**
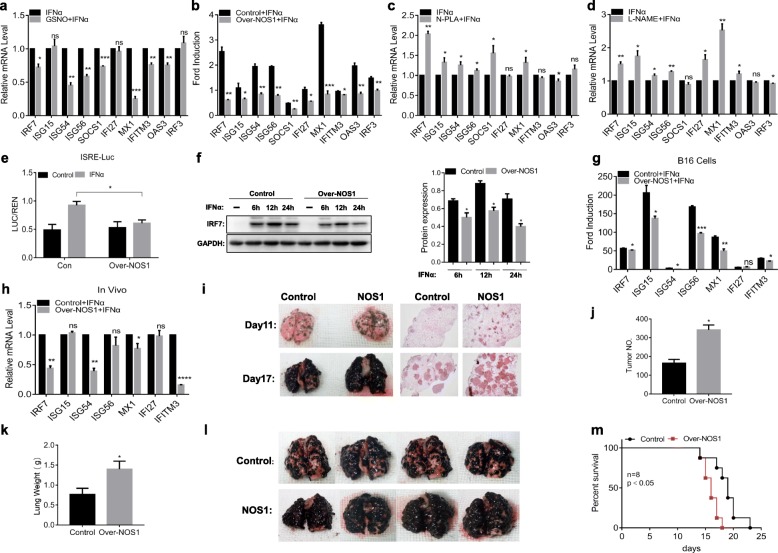
NOS1 blocks IFNα-stimulated gene induction and promotes lung metastasis of melanoma. **a** A375 cells were stimulated with IFNα (1000 U/ml) for 6 h in the presence or absence of simultaneous GSNO (100 μM). The mRNA expression of ISGs was analyzed by RT-PCR. **b** Control/NOS1 (A375) cells were treated with IFNα (1000 U/ml) for 6 h, followed by RT-PCR analysis. **c, d** Similar to **a**, but **c** N-PLA (100 μM), **d** L-NAME (1 mM) was used. **e** Control/NOS1 (A375) cells were cotransfected with pISRE-luc and Renilla-luc reporter plasmids for 24 h, treated with IFNα (1000 U/ml) for 6 h, and analyzed by luciferase assay. **f** Control/NOS1 (A375) cells were incubated with IFNα (1000 U/ml) for the indicated times, and western blotting was used to detect the protein expression of IRF7. **g, h** The effect of Over-NOS1 on the expression of IFNα-ISGs in **g** B16 cells and **h** tumor tissues was detected by RT-PCR. **i** Representative images of lung tissue and lung sections stained with H&E from each group are shown. **j, k** The **j** tumor nodules and **k** lung weight of each group (*n* = 4). **l** Mice were sacrificed individually upon signs of metastatic distress and lung metastasis confirmed via histology. **m** Long rank analysis of mouse survival rates (*n* = 8). ns, not significant; *p < 0.05; **p < 0.01; ***p < 0.001; and ****p < 0.0001

In initial experiments, we examined the role of NO in IFNα-stimulated gene (IFNα-ISG) transcription. We first investigated the response to NO donor GSNO in the melanoma cell line A375 by testing the expression of 10 ISGs, including IRF7, ISG15, ISG54, ISG56, SOCS1, IFI27, MX1, IFITM3, OAS3, and IRF3, by RT-PCR. Treatment of A375 cells with GSNO blocked ISG induction compared to cells treated with IFNα alone (Fig. 1a), and similar ISG suppression was observed in the other two human cancer cell lines SW480 and SKOV3 (Additional file 2: Figure S1a). To confirm that NOS1 inhibited the expression of ISGs and to rule out nonspecific effects of the compound, we stably overexpressed NOS1 (Over-NOS1) in A375, SKOV3 and SW480 cells by lentivirus transfection. The results showed that overexpression of NOS1 significantly reduced the expression of ISGs that we tested compared to nontargeted control cells (Fig. 1b, Additional file 2: Figure S1b). In addition, treatment with a NOS1-specific inhibitor (N-PLA) increased ISG induction of 1–2 ford, and similar ISG expression was observed in a pan-NOS inhibitor (L-NAME) tested in A375 cells (Fig. 1c, d). These results suggest a negative role for NO/NOS1 in the induction of ISGs.

Next, an interferon-sensitive response element (ISRE) luciferase reporter gene assay was carried out to test whether NOS1 is involved in the general transcriptional regulation of ISGF3. Stimulation with IFNα resulted in increased luciferase activity, but Over-NOS1 prevented reporter gene induction (Fig. 1e). The transcription factor IRF7 was considered the master regulator of the type I interferon response; therefore, we further tested the effect of NOS1 on its protein expression. Western blotting results showed that NOS1 reduced the expression of these proteins after IFN stimulation at each time point that we tested (Fig. 1f). These findings support that endogenous NO derived from NOS1 downregulates ISGF3-dependent transcription and gene expression, independent of specific cell lines and genes.

To confirm the results, we also performed a similar study in mouse melanoma cells B16F10, and RT-PCR results showed that NOS1 inhibited the induction of ISGs in both in vivo and in vitro experiments (Fig. 1g, h). Studies have shown that dysfunction of IFN signaling is associated with tumor metastasis [9]. To investigate the role of NOS1-downregulation of IFN signaling in the metastasis of melanoma, we successfully constructed an animal model of melanoma lung metastasis by injecting B16F10-(Control/Over-NOS1) cells into C57BL/6 mice via the tail vein and sacrificed mice on the 11th day after injection to count the number of lung nodules. As shown in Fig. 1i, the number of lung nodules in the Over-NOS1 mice was higher than that in the control mice. On day 17, both lung nodules of each group were significantly increased and could not be counted. On the other hand, we further confirmed that the number of nodules in Over-NOS1 mice was still higher than that in control mice by H&E staining of lung tissue (Fig. 1i). We also measured the lung nodules and lung weight on these 2 days, and the quantification of lung metastases confirmed many metastases in Over-NOS1 mice, whereas in control mice, a few metastases were observed (Fig. 1j, k). There were still more lung nodules in the Over-NOS1 mice when the mice were sacrificed individually upon signs of metastatic distress (Fig. 1l). Moreover, the mean survival times of the control and Over-NOS1 mice were 19 days and 16 days, respectively, and there was a significant difference in survival rates (Fig. 1m). Taken together, these results indicate that NOS1 may promote lung metastasis of melanoma by inhibiting IFN signaling.

### NOS1 inhibits the recruitment of HDAC2 at the promoter of ISGs

**Fig. 2:**
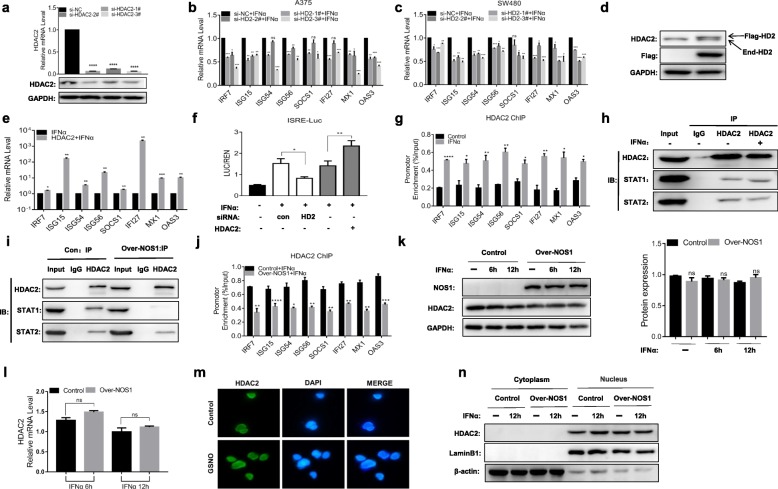
NOS1 inhibits interferon-induced recruitment of HDAC2. **a** Negative control siRNA (si-NC) or an siRNA specific for HDAC2 (si-HDAC2) was used to transfect A375 cells for 48 h, and then the knockdown efficiency was determined by RT-PCR and an immunoblotting assay. **b, c** siRNA was used to transfect **b** A375 cells and **c** SW480 cells for 24 h, followed by stimulation with IFNα (1000 U/ml) for 6 h and RT-PCR analysis. **d** HDAC2 expression plasmids were used to transfect A375 cells for 24 h, and the transfection efficiency was determined by immunoblotting assay. **e** Similar to **b**, HDAC2 expression vectors were used to transfect A375 cells for 24 h before IFNα stimulation. **f** pISRE-luc and Renilla-luc reporter plasmids were used to cotransfect A375 cells in the presence of si-NC (Con), si-HDAC2 (HD2), or HDAC2 expression vectors as indicated. Luciferase activity was measured 24 h after transfection in untreated cells and cells treated with IFNα (1000 U/ml) for 6 h. **g** A375 cells were incubated with or without IFNα (1000 U/ml, 6 h). ChIP-qPCR was used to detect the binding of HDAC2 to the ISG promoters. **h** A375 cells were stimulated with (+) or without (−) IFNα (1000 U/ml) for 6 h, and the lysates were precipitated with HDAC2 or IgG antibodies. Western blotting was performed using the indicated antibody. **i** Similar to **h**, but Co-IP assays were performed in Control/NOS1 (A375) cells. **j** Similar to **g**, ChIP assays were performed in Control/NOS1 (A375) cells by stimulation with IFNα (1000 U/ml) for 6 h. **k**, **l** Control/NOS1 (A375) cells were treated with IFNα (1000 U/ml) for 6 h and 12 h, the expression of HDAC2 was detected by **k** western blotting and **l** RT-PCR. **m** The subcellular localization of HDAC2 was visualized by immunofluorescence staining in A375 cells after treatment with or without GSNO for 6 h. Nuclei were stained with DAPI (blue), original magnification: 100×. **n** A375 cells were treated with or without IFNα (1000 U/ml) for 12 h, and the expression of nuclear and cytoplasmic proteins of HDAC2 was detected by western blotting. ns, not significant; *p < 0.05; **p < 0.01; ***p < 0.001; and ****p < 0.0001. Flag-HD2, Flag-tagged HDAC2 expression vectors; End-HD2, endogenous HDAC2

Previous studies have shown that HDAC2 regulates the expression of ISGs, but the mechanism has not been determined. We further studied this mechanism in melanoma cells. To determine the requirement for HDAC2 for the optimal transcriptional activity of ISGF3, we modified the level of HDAC2 expression by siRNA knockdown or overexpression in A375 cells. The three siRNAs specific for HDAC2 exhibited varied knockdown efficiencies at the mRNA and protein levels by RT-PCR and western blotting, respectively (Fig. 2a). After the knockdown of HDAC2 expression by siRNAs, the mRNA levels of all eight ISGs induced by IFNα were reduced in the HDAC2 siRNA-treated A375 and SW480 cells compared with the scramble-treated control cells (Fig. 2b, c). Because of the difference in interference efficiency, we chose the No.3 siRNA to continue the next experiment. In contrast, the overexpression of HDAC2 by vector transfection significantly increased the transcription of ISGs (Fig. 2d, e). To determine whether HDAC2 is involved in the transcriptional regulation of ISGs, we also performed an ISRE luciferase reporter gene assay. The results showed that the increase of luciferase activity by IFNα stimulation was inhibited by siRNA knockdown of HDAC2 and increased by the overexpression of HDAC2 (Fig. 2f). These results suggested that HDAC2 promotes IFNα-induced transcription in tumor cells.

To understand the mechanism by which HDAC2 promotes the expression of ISGs, we investigated the recruitment of HDAC2 to the promoters of ISGs by ChIP-qPCR with an anti-HDAC2 antibody. The ChIP assay indicated that the binding of HDAC2 with the promoters of ISGs was increased by IFNα stimulation compared to the basal level, suggesting that HDAC2 was recruited to the promoters (Fig. 2g). A prior study reported that activated STAT1 and STAT2 are recruited and bind to HDAC1 to regulate ISG expression [16]. We next investigated the association of HDAC2 with IFNα-activated STAT1 and STAT2 using a co-IP assay. As shown in Fig. 2h, STAT1 or STAT2 expression was found in the cell lysates immunoprecipitated with HDAC2 antibody, which indicates that HDAC2 interacts with both STAT1 and STAT2 (Fig. 2h). This interaction appears unaffected by IFN stimulation, while overexpression of NOS1 in A375 cells inhibited the interaction of HDAC2 with STAT1 but not STAT2 (Fig. 2i). Moreover, overexpression of NOS1 inhibited IFNα-induced recruitment of HDAC2 to the ISG promoters (Fig. 2j).

We asked whether NOS1 inhibits HDAC2 expression or subcellular localization. Therefore, we examined the mRNA and protein expression of HDAC2 in cells overexpressing NOS1. The results showed that NOS1 did not affect the expression of HDAC2 in the presence or absence of IFNα stimulation (Fig. 2k, l, Additional file 2: Figure S2a). The subcellular location of HDAC2 was examined by immunofluorescence assay. Consistent with other reports, HDAC2, stained with FITC, was mostly located in the nucleus of control cells and was not affected by GSNO treatment (Fig. 2m). In addition, we also detected HDAC2 in the cytoplasm and nucleus by western blotting. As Fig. 2n shows, HDAC2 was mainly expressed in the nucleus and was not inhibited by NOS1. These results strongly implicate HDAC2 as a critical positive coactivator for ISGF3-dependent transcriptional responses, and NOS1 inhibits STAT1-mediated recruitment of HDAC2.

### NOS1 induces S-nitrosylation of HDAC2-C262/C274

**Fig. 3:**
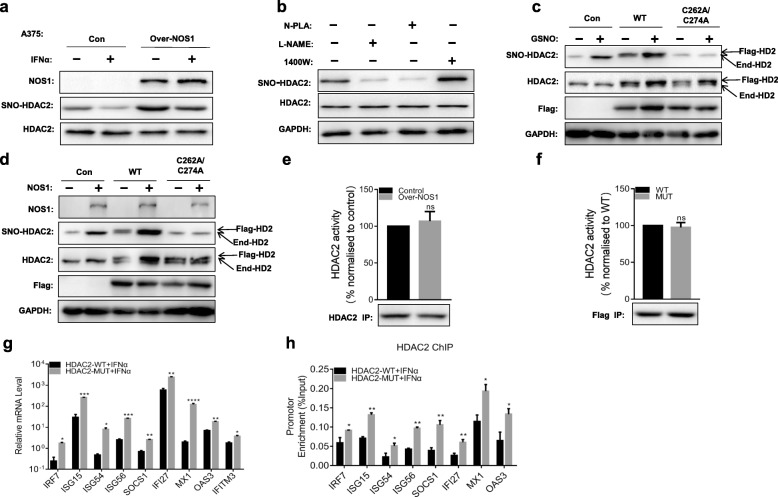
NOS1 induces S-nitrosylation of HDAC2-C262/274. **a** Control/NOS1 (A375) cells were treated with or without IFNα (1000 U/ml) for 6 h. Protein extracts were subjected to the biotin-switch assay. **b** Similar to **a**, but A375 cells were treated with or without L-NAME (1 mM), N-PLA (100 μM), 1400 W (100 μM) for 24 h. **c** Biotin-switch assay of A375 cells transfected with HDAC2-WT, HDAC2-C262A/C274A or empty vector (Control) for 24 h and then treated with GSNO (100 μM, 30 min). **d** Similar to **c**, but Control/NOS1 (A375) cells were used. **e** Control/NOS1(A375) cells were immunoprecipitated HDAC2 was subjected to HDAC activity assay. **f** Over-NOS1 (A375) cells were transfected with the indicated HDAC2 vectors. Flag antibody immunoprecipitates were subjected to HDAC assay. Shown are the averages and SEM (*n* = 3). **g** HDAC2 vectors were transfected into Over-NOS1 (A375) cells, followed by stimulation with IFNα (1000 U/ml) for 6 h and RT-PCR analysis. **h** HDAC2 vectors were transfected into Over-NOS1 (A375) cells and then treated with IFNα (1000 U/ml) for 6 h. HDAC2 immunoprecipitation was followed by ChIP-qPCR analysis. *p < 0.05; **p < 0.01; ***p < 0.001; ****p < 0.0001. Flag-HD2, Flag-tagged HDAC2 expression vectors; End-HD2, endogenous HDAC2

Previous studies reported that HDAC2 was S-nitrosylated by NO in nerve cells, and the S-nitrosylation site was C262/C274 [26]. Therefore, we used the biotin-switch assay to test whether NOS1 directly modifies HDAC2 by means of S-nitrosylation in tumor cells. Under basal conditions, S-nitrosylation of HDAC2 was detected in A375 and SKOV3 cell extracts. Stimulation of cells with IFNα resulted in a small reduction of S-nitrosylation, while overexpression of NOS1 increased this level, even before IFNα stimulation (Fig. 3a, Additional file 2: Figure S2b). To determine whether other NOS subtypes were involved in the S-nitrosylation of HDAC2, A375 cells were treated with NOS1- and NOS2-specific inhibitors (N-PLA, 1400 W) and a pan-NOS inhibitor (L-NAME), respectively. As shown in Fig. 3b, N-PLA and L-NAME were found to induce reduced S-nitrosylation of HDAC2 but not 1400 W (Fig. 3b). This finding suggests that NOS1 but not NOS2 could induce the S-nitrosylation of HDAC2. To further investigate the nitrosylation site of HDAC2, we transferred wild-type HDAC2 (HDAC2-WT) and Cys 262, Cys274 double-mutant (HDAC2-C262A/C274A) plasmids into cells. Compared to transfection of wild-type plasmids, the double mutant form of HDAC2 completely abolished S-nitrosylation of HDAC2 under conditions of GSNO exposure (Fig. 3c), suggesting that C262/C274 is the main S-nitrosylation site of HDAC2. We further conducted a similar study in cells overexpressing NOS1. The results clearly showed that Over-NOS1 significantly increased the S-nitrosylation level of HDAC2 in A375 cells transfected with HDAC2-WT plasmids but not HDAC2-C262A/C274A plasmids (Fig. 3d), indicating that C262/C274 of HDAC2 was the S-nitrosylation site modified by NOS1.

S-nitrosylation of critical cysteine residues may influence HDAC2 enzymatic activity. Therefore, we measured the deacetylase activity of HDAC2 in A375-Control/NOS1 cells. As shown in Fig. 3e, there was no difference in HDAC2 activity between Control and Over-NOS1 cells (Fig. 3e). Moreover, HDAC2 activity was not significantly affected in Over-NOS1 cells expressing HDAC2-WT or HDAC2-C262A/C274A (Fig. 3f). To determine whether the S-nitrosylation of HDAC2 mediates IFNα-dependent transcriptional activation of ISGs in tumor cells, Over-NOS1 (A375) cells were transfected with HDAC2-WT, HDAC2-C262A/C274A plasmids and stimulated with IFNα for 6 h. RT-PCR analysis showed that HDAC2-C262A/C274A increased the induction of ISG expression compared to cells transfected with wild-type plasmids, suggesting that it reversed the inhibitory effect of NOS1 on ISG expression (Fig. 3g). We next analyzed the effect of HDAC2-C262A/C274A on the binding of HDAC2 to the ISG promoter in Over-NOS1 cells. As expected, recruitment of HDAC2-C262A/C274A to chromatin was increased during IFNα stimulation (Fig. 3h). These results indicate that NOS1-dependent S-nitrosylation of HDAC2 on the critical cysteine residues Cys 262 and Cys 274 is necessary to induce the inhibition of ISG expression and recruitment of HDAC2 to chromatin.

### NOS1 inhibits the deacetylation of H4K16 by S-nitrosylation of HDAC2

**Fig. 4:**
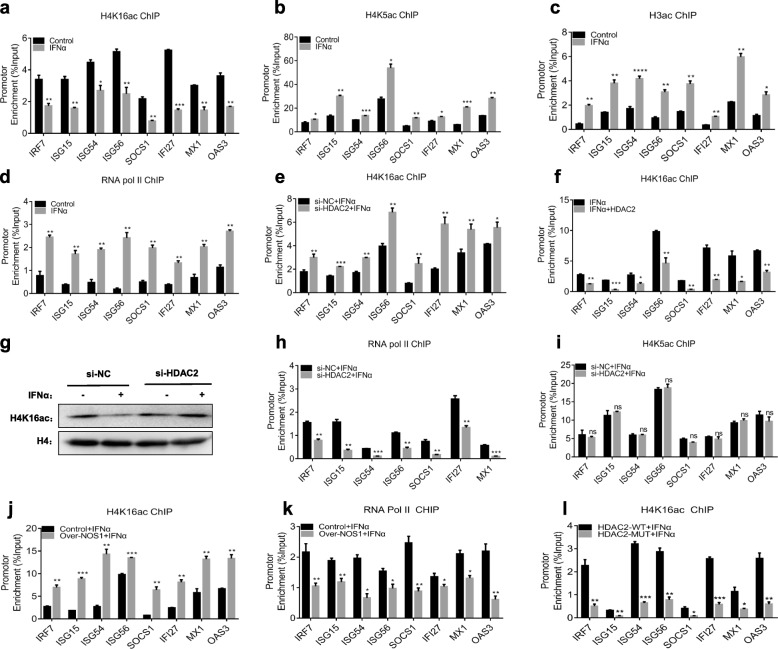
NOS1 inhibits the deacetylation of H4K16 by S-nitrosylation of HDAC2. **a-d** A375 cells were treated with or without IFNα (1000 U/ml) for 1 h. ChIP was performed using specific **a** anti-H4K16ac, **b** anti-H4K5ac, **c** anti-H3ac and **d** anti-Rbp1 antibodies. **e** HDAC2 siRNA or the control siRNA were transfected into A375 cells for 24 h, followed by stimulation with IFNα (1000 U/ml) for 1 h. ChIP was performed using H4K16ac antibodies. **f** Similar to **e**, but HDAC2 expression vectors were used to transfect A375 cells for 24 h before IFNα stimulation. **g** HDAC2 siRNA or control siRNA was used to transfect A375 cells for 24 h and then stimulated with or without IFNα (1000 U/ml) for 6 h. Western blotting was performed using the indicated antibodies. **h, i** Using the same protocol as in **e**, ChIP assays were performed using specific **h** anti-Rpb1 and **i** anti-H4K5ac antibodies. **j, k** Control/NOS1 (A375) cells were treated with IFNα (1000 U/ml) for 1 h. ChIP assays were performed using specific **j** anti-H4K16ac and **k** anti-Rpb1 antibodies. **l** Over-NOS1 (A375) cells were transfected with HDAC2-WT or HDAC2-MUT vectors for 24 h and then treated with IFNα (1000 U/ml) for 1 h. ChIP assays were performed using specific anti-H4K16ac antibodies. ns, not significant; *p < 0.05; **p < 0.01; ***p < 0.001; ****p < 0.0001

The regulation of the specific-site acetylation status of histone is critical for maintaining IFN signal integrity. Histone H4 becomes deacetylated in the ISGF3 target promoter ISG54 in response to IFNα [16], and the acetylation of H4K16 was inconsistent with that of three other residues (H4K5, H4K8, and H4K12) [32]. H4K16 acetylation (H4K16ac) plays a distinct role in gene silencing [33]. Therefore, we analyzed the acetylation status of histone H4K16 in response to IFNα stimulation by ChIP assays. As shown in Fig. 4a, basal H4K16 acetylation at the promoters of the ISGs was detected readily but was reduced significantly after IFNα stimulation (Fig. 4a). Consistent with other published reports, the acetylation levels of H4K5 and H3 were significantly increased by IFNα stimulation as measured by ChIP assay (Fig. 4b, c). RNA pol II is a crucial regulator of initiation of transcription, and it was previously reported that HDAC activity is required for recruitment of RNA pol II to the promoter of ISGs. Therefore, we performed ChIP analysis with an anti-RNA pol II antibody (Rpb1) to evaluate the transcriptional initiation of ISGs. As Fig. 4d shows, the binding of RNA pol II to the promoters was increased after IFNα stimulation (Fig. 4d). This indicates that in contrast to the increased acetylation levels of H4K5 and H3, H4K16 is deacetylated in response to IFNα stimulation, which might be beneficial to the recruitment of RNA polymerase II for the expression of ISGs.

We next evaluated the involvement of HDAC2 in H4K16 deacetylation at the promoters of ISGs after IFNα stimulation. The levels of H4K16 acetylation increased by HDAC2 siRNA compared with the “scrambled” control (Fig. 4e). In contrast with this result, the overexpression of HDAC2 decreased the binding of acetylated H4K16 at the ISG promoters (Fig. 4f). Furthermore, the protein levels of acetylated H4K16 were decreased by IFNα treatment, while si-HDAC2 further increased the level of H4K16ac (Fig. 4g, Additional file 2: Figure S3a). si-HDAC2 also reduced the recruitment of RNA pol II to all ISG promoters (Fig. 4h). However, the acetylation statuses of H4K5 and H3 bound to the promoters of ISGs were not altered by si-HDAC2 (Fig. 4i, Additional file 2: Figure S3b). These results show that HDAC2 regulates the acetylation status of H4K16 at the ISG promoters, which may facilitate the recruitment of RNA pol II to ISG promoters.

We further analyzed the involvement of NOS1 in the H4K16ac status at the ISG promoter by ChIP assay. The results showed that Over-NOS1 increased the acetylation of H4K16 in the promoter of all ISGs we detected, and inhibited the recruitment of RNA pol II to the promoter (Fig. 4j, k). When Over-NOS1 cells were transfected with HDAC2-C262A/C274A plasmids, IFNα failed to induce H4K16 acetylation of the chromatin surrounding all 8 ISG promoters (Fig. 4l). This finding indicates that NOS1 inhibits the deacetylation of H4K16 by Snitrosylation of HDAC2-C262/C274.

### NOS1 promotes melanoma lung metastasis by S-nitrosylation of HDAC2-C262/274

**Fig. 5:**
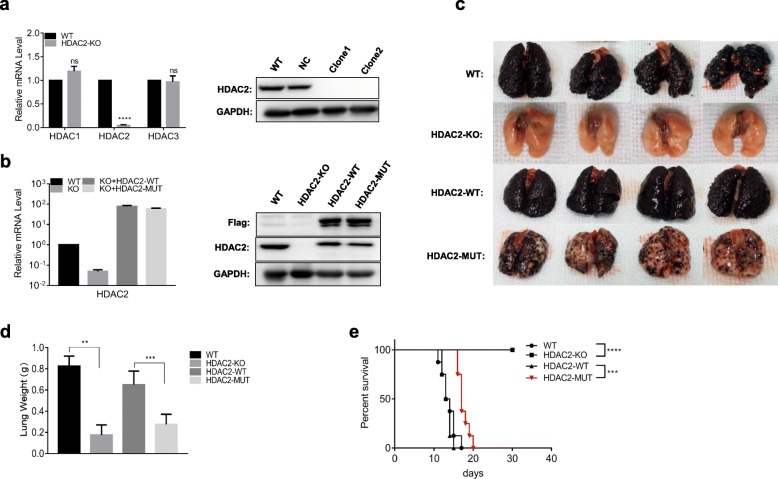
NOS1 promotes melanoma lung metastasis by S-nitrosylation of HDAC2-C262/274. **a** The mRNA expression of HDACs (1, 2, 3) and protein expression of HDAC2 in HDAC2-KO cells were determined by RT-PCR and western blotting. **b** The same method as **a** was used to detect the mRNA and protein expression levels of HDAC2 of stably transfected HDAC2-WT and HDAC2-MUT plasmids in HDAC2-KO cells. **c-e** 3 × 10^6^ B16-WT, B16-HDAC2-KO, B16-HDAC2-WT, B16-HDAC2-MUT cells were intravenously injected into C57BL/6 mice. The mice were sacrificed on the 11th day after tumor cell inoculation. Tumor growth was monitored by **c** gross morphology, **d** lung weight (*n* = 4), and **e** survival rate (*n* = 8). ns, not significant; **p < 0.01; ***p < 0.001; ****p < 0.0001

In previous experiments, we could not rule out the effect of endogenous HDAC2 on lung metastasis; therefore, we further constructed a mouse melanoma (B16F10-NOS1) cell line that knocked out HDAC2 using the CRISPR/Cas9 gene editing method. The protein and mRNA expression of HDAC2 in knockout cells were detected by western blotting and RT-PCR. As shown in Fig. 5a, the expression of HDAC2 in the KO cells was significantly reduced, but it had no significant effect on the expression of HDAC1 and HDAC3 (Fig. 5a). This result indicates that we have successfully constructed the B16F10-HDAC2-KO cell lines. Subsequently, we also constructed cell lines stably expressing HDAC2-WT and HDAC2-C262A/C274-MUT in HDAC2-KO cells, and the results showed that the protein and mRNA expression of HDAC2 was restored (Fig. 5b).

We next investigated the metastasis of melanoma in vivo. An animal model was constructed by injecting 3 × 10^6^ cells into the mice by tail vein, and the mice were divided into four groups: B16-WT, B16-KO, B16-KO + HDAC2-WT and B16-KO + HDAC2-MUT. The mice were sacrificed on the 11th day after injection to obtain lung tissue. As Fig. 5c shows, the number of lung nodules in the B16-KO mice was significantly lower than that of B16-WT mice, and no obvious tumor was observed. In contrast, after restoring the expression of HDAC2, the lung nodules in the B16-KO + HDAC2-WT mice were similar to that of B16-WT mice, but significantly increased compared with B16-KO mice (Fig. 5c). This finding suggests that knockout of HDAC2 in B16 cells significantly inhibited lung metastasis, whereas stable expression of HDAC2-WT/MUT in KO cells restored lung metastasis of melanoma. Compared with HDAC2-WT, the mice of HDAC2-MUT had a lower lung metastasis, and the lung weight was significantly decreased (Fig. 5d). The survival time of HDAC2-MUT mice was longer than that of HDAC2-WT mice, indicating that the HDAC2 mutation partially reversed the promotion of NOS1 on lung metastasis of melanoma (Fig. 5e). These data imply that NOS1 promotes melanoma lung metastasis by S-nitrosylation of HDAC2-C262/274.

### NOS1 inhibits tumor lymphocyte infiltration by S-nitrosylation of HDAC2-C262/274

**Fig. 6:**
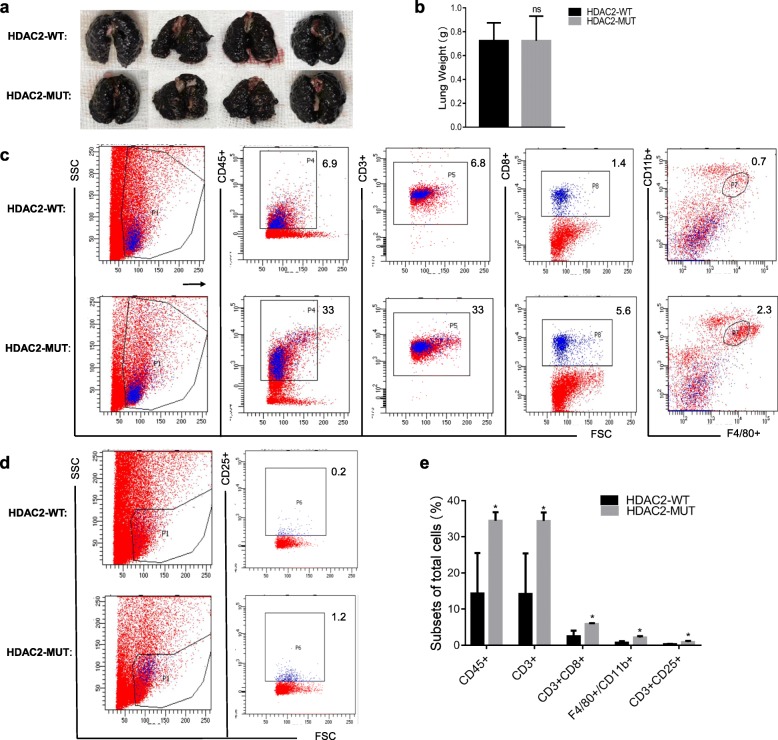
NOS1 inhibits tumor lymphocyte infiltration by S-nitrosylation of HDAC2-C262/274. **a, b** B16-HDAC2-WT, B16-HDAC2-MUT cells were intravenously inoculated into BALB/c-nu mice (3 × 10^6^/mouse). The mice were sacrificed on the 11th day after inoculation. Tumor growth was monitored by **a **gross morphology and **b** lung weight (n = 4)**. c-e** Analysis of lymphocyte cell subsets and activation status by flow cytometry. B16-HDAC2-WT or B16-HDAC2-MUT cells were intravenously injected into each C57BL/6 mouse (3 × 10^6^/mouse). The mice were sacrificed on the 11th day, and the lungs were isolated. The infiltration of various immune cell populations into the tumors was evaluated. Tumor cells from lung were stained for **c** CD45, CD3, CD8, F4/80, CD11b and **d** CD25 followed by flow cytometry analysis. Representative flow cytometry plots are shown, and **e** quantification is of data from 3 animals in each group. ns, not significant; * *P* < 0.05

IFNα has been reported to have both anti-proliferative and immunomodulatory effects. Because we found that HDAC2-MUT partially reversed the role of NOS1 in promoting lung metastasis, we further investigated whether HDAC2-MUT directly inhibited tumor growth. In the in vitro experiment, we did not observe differences in the growth of HDAC2-WT and HDAC2-MUT cells; therefore, we injected the cells into BALB/c-nu mice. Lung metastasis was observed on the 11th day after cell injection, and the results showed that there was no significant difference between the two groups (Fig. 6a, b). This result indicates that HDAC2-MUT does not directly inhibit tumor growth. A possible explanation is that HDAC2-MUT reduces lung metastasis by regulating antitumor immunity. Therefore, we injected HDAC2-WT and HDAC2-MUT cells into C57BL/6 mice, and the isolated lung tissues were detected by flow cytometry. As shown in Fig. 6c, the lung from HDAC2-WT mice contained significantly reduced numbers of CD45+, CD3+, CD3 + CD8+ T cells and F4/80 + CD11b + macrophages compared to lung from HDAC2-MUT mice (Fig. 6c, e). We also examined the number of activated T cells in the lungs of both groups. Similarly, the lungs of HDAC2-MUT mice contained elevated numbers of CD3 + CD25+ T cells (Fig. 6d, e). Taken together, these results indicate that NOS1-induced S-nitrosylation of HDAC2 promotes lung metastasis primarily by inhibiting tumor lymphocyte infiltration.

## Discussion

The expression of NOS1 in melanoma is closely correlated with dysfunctional type I IFN signaling and poor prognosis of patients. However, the underlying mechanism governing this role has not been determined. In this study, we investigated the regulation of NOS1 on IFN signaling and lung metastasis. Our findings are as follows: (i) HDAC2 upregulates ISG expression by deacetylating H4K16, increasing the recruitment of RNA polymerase II to the promoter; and (ii) NOS1 reduces STAT1-mediated recruitment of HDAC2 to the ISG promoter and deacetylation of H4K16 by S-nitrosylation of HDAC2-C262/C274, which results in inhibition of IFN signaling and tumor lymphocyte infiltration, thereby promoting lung metastasis of melanoma.

ISGs remain silent under normal conditions, but de novo transcription is initiated by activated transcription factors after stimulation by IFNα. The class I HDAC family has often been associated with the suppression of gene transcription via repressive complexes, while it acts as activators of gene expression in IFN-induced STAT1-dependent transcription [18]. Consistent with previous reports, our results also showed that the expression and activity of HDAC2 were positively correlated with ISG transcription in melanoma cells. In this study, the inhibition of HDAC2 with an siRNA decreased IFNα responsiveness, while the overexpression of HDAC2 augmented the IFNα response of tumor cells (Fig. 2b-f). We further revealed that HDAC2 was recruited to the promoters of ISGs by STAT1 and STAT2 after IFNα stimulation (Fig. 2g, h). IFNα treatment induced the acetylation of histones H4K5 and H3 but was accompanied by the deacetylation of H4K16 at the promoters of ISGs. Furthermore, the modulation of HDAC2 expression by siRNA-mediated knockdown and overexpression decreased and increased, respectively, the deacetylation level of histone H4K16 but had no impact on the acetylation status of histones H4K5 and H3. In addition, IFNα also induced the recruitment of RNA pol II to the promoter, which was inhibited by si-HDAC2, indicating that HDAC2 is required for the recruitment of RNA pol II (Fig. 4).

Acetylation of lysine residues of histone H3 and histone H4 are critical for maintaining the integrity of IFN signals [34]. Specifically, acetylation of H3 is considered a marker of general transcriptional activation. STAT2 has been reported to recruit the histone acetyltransferase GCN5 to promote acetylation of H3 after IFNα stimulation [35]. There are four acetyl-lysine residues, K5, K8, K12 and K16, in H4, but the generation and properties of acetylated histone H4K16 are distinct from those of other acetylated sites in H4 (i.e., K5, K8, and K12) [36]. For instance, the mutation of K5, K8 or K12 results in similar effects and positively correlates with gene transcription, and these three sites complement each other. In contrast, H4K16 acetylation is not correlated with the other sites and is negatively associated with gene expression [32]. Acetylated H4K16 is a key epigenetic marker involved in gene regulation and chromatin remodeling [37, 38]. Although this marker is known to be essential for embryonic development and heterochromatin formation [33, 39], its role during ISG expression has not been determined. Previous studies confirmed that viral infection induces significant H4K8 and H4K12 acetylation at the IFNβ promoter, while H4K16 is not acetylated during transcriptional activation [40]. In this study, we show evidence of H4K16 deacetylation by HDAC2 under IFNα stimulation conditions in melanoma cells (Fig. 4e-g), providing a molecular mechanism of HDAC2 involvement in the regulation of ISG expression through histone modification. The observed H4K16 deacetylation, accompanied by an increase in the recruitment of RNA pol II (Fig. 4d, h), suggests that HDAC2 is a transcriptional regulator for ISGs through chromatin remodeling, transcription-activating complex recruitment, and transcription initiation. HDAC1 has been reported to play an essential role in IFNα-induced transcription, but whether HDAC1 works similarly to HDAC2 to deacetylate H4K16 for ISG expression was not investigated in this study. Our study reveals the positive role of HDAC2 in the IFNα response and provides new insights into the epigenetic regulation of IFNα signaling. Nevertheless, the induced expression process of ISGs is regulated by a series of factors, and a comprehensive understanding of its regulatory mechanisms requires more in-depth research.

In recent years, the role of nitric oxide in tumor biology has received increasing attention [41, 42]. NOS1 produces constitutively low levels of NO, which generally promote tumor growth, such as cell proliferation, anti-apoptosis and migration, in many cellular processes [21]. Meanwhile, it has been observed that NOS1 expression and increased NO production correlate with a poor prognosis in melanoma patients [43]. Furthermore, NOS1 was closely related to dysfunctional IFN signaling, and an inhibitor of NOS1 resulted in reduced proliferation of melanoma cells [25, 44]. In this study, we found that endogenous NO from NOS1 downregulates ISGF3-dependent transcription and gene expression. On the other hand, animal models indicate that NOS1 promotes lung metastasis of melanoma (Fig. 1). These results further provide evidence that NOS1 is involved in dysfunctional IFN signaling and melanoma metastasis. NO is a molecule capable of modifying cysteines by S-nitrosylation, which affects protein function by altering the interaction between proteins, subcellular localization or catalytic activity [24]. Previous studies have shown that S-nitrosylation of HDAC2-C262/C274 does not alter the enzyme’s catalytic activity but induces its release from chromatin [26, 45]. HDACs exist as components of multiprotein complexes amd are then targeted to specific genomic regions by interactions with DNA binding factors such as transcription factors [46]. Furthermore, histone hyperacetylation is not always the result of a loss of HDAC activity, but it could be due to a loss of HDAC targeted to specific DNA sequences [47]. We further demonstrated that NOS1-induced S-nitrosylation reduces the recruitment of HDAC2 to the ISG promoter by inhibiting the interaction of HDAC2 with STAT1 (Fig. 2i, j, and Fig. 3h). This effect resulted in increased acetylation of H4K16 (Fig. 4l) and transcriptional inhibition of ISGs (Fig. 3g). Thus, our study demonstrated a linkage between the S-nitrosylation of HDAC2 and the regulation of ISG expression, providing an endogenous NO-mediated mechanism for the dysfunction of the IFNα response in melanoma cells.

The loss of tumor cell type I IFN production, and hence immune signalling, was associated with an increased risk of metastasis [48]. Downregulation of IRF7 has been described in breast cancer and contributes to tumor metastasis, indicating that IFN signaling is involved in the control of metastatic spread [9]. Our study confirmed that NOS1 promotes lung metastasis at least partly through S-nitrosylation of HDAC2-C262/C274 and deregulation of the IFNα response. Interestingly, in animal models, lung metastasis was significantly reduced after knockout of HDAC2 in melanoma cells (Fig. 5c), suggesting that HDAC2 is a tumor-promoting factor, and other studies also support this finding [49–51]. There is more evidence for HDAC2 overexpression in certain cancers compared with normal tissues [52]. HDAC2 regulates the cell cycle and apoptosis of cancer cells, and gene editing of HDAC2 resulted in a more differentiated phenotype and increased apoptosis caused by augmented levels of p21 [53]. HDAC2 also plays a role in controlling cell survival by regulating p53 and its target genes [54]. Transformation of cells could be caused by elevated HDAC2, for example via inactivation of p53 or regulation of p53-DNA binding activity [55]. Thus, HDAC2 appears to represent a therapeutic target, and the development of anticancer drugs specific for HDAC2 may inhibit tumor metastasis and prevent the side effects from the current pan-HDACi treatment. Importantly, HDAC2 bearing a mutation of Cys262 and Cys274 (HDAC2-C262A/274A) cannot be nitrosylated and acts as a potent transcriptional activator in the IFNα pathway, thereby reversing the inhibition of ISGs and promotion of lung metastasis by NOS1.

Type I IFNs have emerged as central coordinators of tumor–immune-system interactions, including stimulation of anti-tumor effector cells (T cells, NK cells, dendritic cells), and negative regulation of suppressive cells (MDSCs and Treg cells) [56]. Recent studies have shown that the expression and secretion of tumor-inherent IFNs is a key player in the anti-tumor immune cascade, influencing the immunogenicity, progression and therapeutic response of tumors [57]. Unfortunately, impaired interferon signaling has been reported to be a common defect in human cancer [3], but the mechanisms underlying tumor-inherent IFN dysfunction have not been determined. In this study, we found that NOS1 modifies HDAC2 by S-nitrosylation, resulting in increased tumor metastasis in B16F10 mice. However, we did not find evidence that NOS1 promotes proliferation in vitro, and the effect of NOS1 was completely abolished in immunocompromised nude mice (Fig. 6a). This finding suggests that the mechanism of metastasis promotion by NOS1 expression in tumor cells is caused by inhibition of tumor immune surveillance. Notably, mice bearing HDAC2-C262A/C274A tumors had an increase in the number of tumor infiltrating lymphocytes (TILs), such as CD8+ T cells and macrophages, which were linked with the type I IFN pathway (Fig. 6c). TILs are known to be key players in antitumor immunity, and their intratumoral accumulation is associated with favorable outcomes in many cancers [58]. In this study, we report for the first time that S-nitrosylation of HDAC2 inhibits IFN signaling and the accumulation of immune cells, impeding the suppression of metastases by T lymphocytes and macrophages. These results suggest that the detection of HDAC2-C262/C274 S-nitrosylation can be used as a marker to determine the IFN treatment response and cancer prognosis.

## Conclusions

We report the mechanism by which HDAC2 regulates the expression of ISGs in tumor cells, and NOS1 induces epigenetic changes through S-nitrosylation of HDAC2, thereby leading to dysfunctional IFN signaling and promoting lung metastasis. These results show that by inhibiting the S-nitrosylation of HDAC2 in tumor cells, IFN signaling can be restored to inhibit metastasis. Our data will prompt future research to target NOS1 or combined immunotherapy to control metastasis in melanoma patients.

## Supplementary information

**Additional file 1: **Sequences of siRNA and primer used in this study. **Table S1.** Primer sequences for RT-PCR. **Table S2.** The sequences of siRNA. **Table S3.** Primer sequences for ChIP-qPCR

**Additional file 2: Figure S1.** NOS1 blocks IFNα-stimulated gene induction. **a** SKOV3 and SW480 cells were treated with IFNα for 6 h in the presence or absence of simultaneous GSNO. The mRNA expression of ISGs were analyzed by RT-PCR. **b** Control/NOS1 (SKOV3, SW480) cells were incubated with IFNα for 6 h, followed RT-PCR analysis. **Figure S2.** S**-**nitrosyltion of HDAC2 does not affect its expression. **a** Control/NOS1 (SKOV3) cells were treated with IFNα (1000 U/ml) for 6 h and 12 h, the expression of HDAC2 was detected by RT-PCR and western blotting. **b** Control/NOS1 (SKOV3, B16) cells were stimulated with or without IFNα for 6 h. Protein extracts were subjected to the biotin-switch assay. **Figure S3.** HDAC2 regulates the acetylation status of H4K16. **a** Densitometric analysis of the data in Fig. 4g (n=3). **b** A375 cells were transfected si-RNA for 24 h and treatment with IFNα for 1 h. ChIP assays were performed after chromatin was immunoprecipitated with an anti-H3ac antibody. IP chromatin was subjected to qPCR. ns, not significant.

## Data Availability

The datasets used and/or analysed during the current study are available from the corresponding author on reasonable request.

## Abbreviations

IFN: Interferon
NOS1: Nitric Oxide Synthase 1
ISGs: Interferon-stimulated genes
HDAC2: Histone deacetylase 2
H4K16ac: Acetylated H4 lysine 16
H4K5ac: Acetylated H4 lysine 5
H3ac: Acetylated H3
RNA pol II: RNA Polymerase II
RNA: Polymerase II
ISGF3: Interferon -stimulated gene factor 3
ISRE: Interferon-sensitive response element
NO: Nitric oxide
PBMCs: Peripheral blood mononuclear cells
STAT: Signal transducer and activator of transcription
IRF: Interferon regulatory factor
ChIP: Chromatin immunoprecipitation
TILs: Tumor infiltrating lymphocytes
