# Supplementary material for: NOS1 inhibits the interferon response of cancer cells by S-nitrosylation of HDAC2
Source: J Exp Clin Cancer Res. 2019 Dec 5;38:483. doi: 10.1186/s13046-019-1448-9 (PMC6896289; doi:10.1186/s13046-019-1448-9)
Supplement: Supplementary file 1 — Additional file 1: Sequences of siRNA and primer used in this study. Table S1. Primer sequences for RT-PCR. Table S2. The sequences of siRNA. Table S3. Primer sequences for ChIP-qPCR [file 13046_2019_1448_MOESM1_ESM.docx]

**Supplementary tables.** Sequences of siRNA and primer used in this study.

**Supplementary table 1.** Primer sequences for RT-PCR

| Gene symbol | Sense primer(5’-3’) | Antisense primer(5’-3’) |
| --- | --- | --- |
| GAPDH (H) | CTCCAAAATCAAGTGGGGCG | TGGTTCACACCCATGACGAA |
| IRF7 (H) | ATGGGCAAGTGCAAGGTGTA | GATGGTATAGCGTGGGGAGC |
| ISG15 (H) | GCGCAGATCACCCAGAAGAT | GTTCGTCGCATTTGTCCACC |
| ISG54 (H) | GAGCAGCCTACGGCAACTAA | GCCTCGTTTTGCCCTTTGAG |
| ISG56 (H) | GTGCTTGAAGTGGACCCTGA | CCTGCCTTAGGGGAAGCAAA |
| SOCS1 (H) | GTAGGAGGTGCGAGTTCAGG | GACCCCTTCTCACCTCTTGA |
| IFI27 (H)  MX1 (H)  IFITM3 (H)  OAS3 (H)  IRF3 (H)  HDAC2 (H)  GAPDH (M)  IRF7 (M)  ISG15 (M)  ISG54 (M)  ISG56 (M)  MX1 (M)  IFI27 (M)  IFITM3 (M)  HDAC1 (M)  HDAC2 (M)  HDAC3 (M) | GCCACAACTCCTCCAATCAC  AGGCAAGGTCAGTTACCAGG  ACACTGTCCAAACCTTCTTCTCT  CTCTCAAGGGTGGCTGTGAT  AGGATGCACAGCAGGAGGAT  GCCACTGCCGAAGAAATGAC  CTACCCCCAATGTGTCCGTC  TGAGCGAAGAGAGCGAAGAG  ACGATTTCCTGGTGTCCGTG  CACTGGAGAGCAATCTGCGA  TGTCATTCGCTATGCAGCCA  CCCCAGAGGCAGTGGTATTG  CCCATTGGAGCCATGACAGT  GAGGACAGCCCCCAAACTAC  ACCACCAGAGGGTGCTCTAT  TTTTGTCAGCTCTCCACGGG  TGGTGGACTTCTACCAGCCG | ATCAGCAGTGACCAGTGTGG  CACCAGATCAGGCTTCGTCA  GTTGAACAGGGACCAGACGA  GATCTACGGATGTCAGGCGG  TGTCTGGCTGGGAAAAGTCC  TCCAGCCCAATTAACAGCCA  TGAAGTCGCAGGAGACAACC  CCAGTAGATCCAAGCTCCCG  CTCGCTGCAGTTCTGTACCA  GCCAGTCATCCAGACGGTAG  CATGAATGGCCTGTTGTGCC  GCCTACCCCAGCAATGAAGT  TAGATGGCACGGAGAGTCCA  CTCCAGTCACATCACCCACC  ATTCGTCGTCAATGCCGTCT  GAAACAACCAAGCCTGTCCC  ATATGTCCAACACCGGGCAA |

**Supplementary table 2.** The sequences of siRNA

| siRNA | Sense primer(5’-3’) | Antisense primer(5’-3’) |
| --- | --- | --- |
| si-NC | UUCUCCGAACGUGUCACGUTT | ACGUGACACGUUCGGAGAATT |
| si-HDAC2-1# | CCCAUAACUUGCUGUUAAATT | UUUAACAGCAAGUUAUGGGTT |
| si-HDAC2-2# | GCAAAUACUAUGCUGUCAATT | AUAUUCUGGAGUGUUCUGGTT |
| si-HDAC2-3# | CCAGAACACUCCAGAAUAUTT | GCCTCGTTTTGCCCTTTGAG |

**Supplementary table 3.** Primer sequences for ChIP-qPCR

| Gene symbol | Sense primer(5’-3’) | Antisense primer(5’-3’) |
| --- | --- | --- |
| IRF7 | GTCCACCTCCCATTACCCAC | GGACGGGAAGTTTCGTCTCG |
| ISG15 | ACTGCCCTAAACCGAGTGTTG | GGGAAAAGCAAAAGTGGCGG |
| ISG54 | TTCCCCTCTGATCACTTTGGTT | CGACAGGGCAGGGGATTTTA |
| ISG56 | ACGGCTGCCTAATTTACAGCAA | TCACCATTTGTACACATCTCCACT |
| SOCS1 | GGCTCCTCACATGCCTTCAT | CCCGACCCCTTCCTCATAGA |
| IFI27  MX1  OAS3 | CATCTGCCTATCGCAAGGACT  CTTTCTGGAAACCAGCGAGCA  AGCGAGGTAGGACTTCTCCG | GGATGGGAGTGTGCTCTACG  CGGTGCTTCTGGAATGAATGG  CTTGGACCTGACACCCACTT |
