# Supplementary material for: NOS1 inhibits the interferon response of cancer cells by S-nitrosylation of HDAC2
Source: J Exp Clin Cancer Res. 2019 Dec 5;38:483. doi: 10.1186/s13046-019-1448-9 (PMC6896289; doi:10.1186/s13046-019-1448-9)
Supplement: Supplementary file 2 — Additional file 2: Figure S1. NOS1 blocks IFNα-stimulated gene induction. a SKOV3 and SW480 cells were treated with IFNα for 6 h in the presence or absence of simultaneous GSNO. The mRNA expression of ISGs were analyzed by RT-PCR. b Control/NOS1 (SKOV3, SW480) cells were incubated with IFNα for 6 h, followed RT-PCR analysis. Figure S2. S-nitrosyltion of HDAC2 does not affect its expression. a Control/NOS1 (SKOV3) cells were treated with IFNα (1000 U/ml) for 6 h and 12 h, the expression of HDAC2 was detected by RT-PCR and western blotting. b Control/NOS1 (SKOV3, B16) cells were stimulated with or without IFNα for 6 h. Protein extracts were subjected to the biotin-switch assay. Figure S3. HDAC2 regulates the acetylation status of H4K16. a Densitometric analysis of the data in Fig. 4g (n=3). b A375 cells were transfected si-RNA for 24 h and treatment with IFNα for 1 h. ChIP assays were performed after chromatin was immunoprecipitated with an anti-H3ac antibody. IP chromatin was subjected to qPCR. ns, not significant. [file 13046_2019_1448_MOESM2_ESM.zip › Supplementary Figure legend.docx]

**Figure S1.** NOS1 blocks IFNα-stimulated gene induction. **a** SKOV3 and SW480 cells were treated with IFNα for 6 h in the presence or absence of simultaneous GSNO. The mRNA expression of ISGs were analyzed by RT-PCR. **b** Control/NOS1 (SKOV3, SW480) cells were incubated with IFNα for 6 h, followed RT-PCR analysis.

**Figure S2.** S**-**nitrosyltion of HDAC2 does not affect its expression. **a** Control/NOS1 (SKOV3) cells were treated with IFNα (1000 U/ml) for 6 h and 12 h, the expression of HDAC2 was detected by RT-PCR and western blotting. **b** Control/NOS1 (SKOV3, B16) cells were stimulated with or without IFNα for 6 h. Protein extracts were subjected to the biotin-switch assay.

**Figure S3.** HDAC2 regulates the acetylation status of H4K16. **a** Densitometric analysis of the data in Fig. 4g (n=3). **b** A375 cells were transfected si-RNA for 24 h and treatment with IFNα for 1 h. ChIP assays were performed after chromatin was immunoprecipitated with an anti-H3ac antibody. IP chromatin was subjected to qPCR. ns, not significant.
